# Moment closure of infectious diseases model on heterogeneous metapopulation network

**DOI:** 10.1186/s13662-018-1801-x

**Published:** 2018-09-24

**Authors:** Shanshan Feng, Zhen Jin

**Affiliations:** 1 School of Data Science and Technology, North University of China, Taiyuan, China; 2 Complex Systems Research Center, Shanxi University, Taiyuan, China; 3 Shanxi Key Laboratory of Mathematical Techniques and Big Data Analysis on Disease Control and Prevention, Shanxi University, Taiyuan, China; 4 Key Discipline of Computer Science and Technology of “Double-First-Class” Project of Shanxi Province, Shanxi University, Taiyuan, China

**Keywords:** Metapopulation network, Infectious diseases, Marcov process, Moment closure

## Abstract

The global transmission of infectious diseases poses huge threats to human. Traditional heterogeneous mean-field models on metapopulation networks ignore the heterogeneity of individuals who are in different disease states in subpopulations with the same degree, resulting in inaccuracy in predicting the spread of disease. In this paper, we take heterogeneity of susceptible and infectious individuals in subpopulations with the same degree into account, and propose a deterministic unclosed general model according to Markov process on metapopulation networks to curve the global transmission of diseases precisely. Then we make the general model closed by putting forward two common assumptions: a two-dimensional constant distribution and a two-dimensional log-normal distribution, where the former is equivalent to the heterogeneous mean-field model, and the latter is a system of weighted ordinary differential equations. Further we make a stability analysis for two closed models and illustrate the results by numerical simulations. Next, we conduct a series of numerical simulations and stochastic simulations. Results indicate that our general model extends and optimizes the mean-field model. Finally, we investigate the impacts of total mobility rate on disease transmission and find that timely and comprehensive travel restriction in the early stage is an effective prevention and control of infectious diseases.

## Introduction

In recent years, global transmission of infectious diseases, such as severe acute respiratory syndromes (SARS) [1], influenza A (H1N1) flu [2], avian influenza [3], Middle East respiratory syndrome coronavirus (MERS-CoV) [4], Ebola virus disease [5], and zika [6], has been threatening human beings. Great attention has been paid to the effects of human mobility on disease transmission.

**Figure 1:**
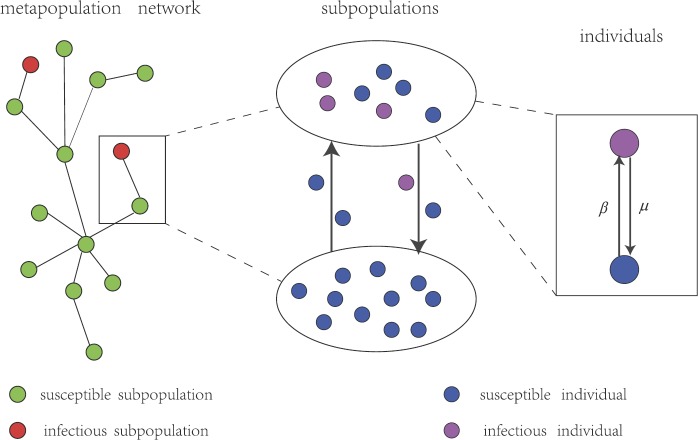
A metapopulation network model of SIS infections with individuals’ mobility. The model is composed of a heterogeneous network of subpopulations, connected by mobility processes. Individuals in each subpopulation stay one of the two states: one is susceptible; the other is infectious. Individuals can move from a subpopulation to another along links of network. Once there exist infectious individuals in a subpopulation, it becomes infectious. For infectious diseases, a susceptible individual can be infected at rate *β* and become infectious, while an infectious individual may recover at rate *μ* and be susceptible

In order to study the global spread of infectious diseases, researchers applied a metapopulation model to infectious diseases [7, 8]. The concept of metapopulation was put forward by Levins [9, 10] for the first time to investigate the processes of local extinction, recolonization and regional persistence of populations, which means “the population of populations”. With the development of network transmission dynamics, the metapopulation model has been successfully applied to understand the transmission dynamics of spatially structured populations with well-defined social units (on a scale of countries, regions, cities, small as towns, villages, families) connected through individuals’ mobility on networks, named “metapopulation networks” (see Fig. 1).

In general, we consider susceptible-infectious-susceptible (SIS) transmission process on a metapopulation network with *V* nodes, label the nodes with the elements of integer set $A=\{1,\ldots,V\}$, and denote by $S_{j}^{k}$ and $I_{j}^{k}$ the number of susceptible and infectious individuals in a node which gets label *j* and degree *k*, respectively. The total individuals of node whose label is *j* and degree is *k* is $N_{j}^{k}$ and $N_{j}^{k}=S_{j}^{k}+I_{j}^{k}$. Then the sum of susceptible and infectious individuals of all nodes with degree *k* are

$$\sum_{j\in A} S_{j}^{k},\qquad \sum_{j\in A} I_{j}^{k}, $$

respectively. Let $V_{k}^{*}$ denote the number of nodes with degree *k*. According to heterogeneous mean-field (HMF) theory, assuming that subpopulations with the same degree are statistical equivalence, that is to say, the average number of individuals in all nodes with degree *k* is

$$ N_{k}=\frac{1}{V_{k}^{*}}\sum_{j\in A} N_{j}^{k}. $$

Further, the average numbers of susceptible and infectious individuals in nodes with degree *k* are

1.1 $$ S_{k}=\frac{1}{V_{k}^{*}}\sum_{j\in A} S_{j}^{k},\qquad I_{k}=\frac{1}{V_{k}^{*}}\sum _{j\in A} I_{j}^{k}, $$

respectively.

Based on the HMF assumption, Colizza and Vespignani [11] proposed some models to address the transmission of diseases on heterogeneous metapopulation networks under two different mobility patterns. They assumed that the process of diseases spreading was in the first, and mobility process next. For the mobility process, at each time step, an individual in a subpopulation with degree *k*, whether susceptible or infectious, travels to other subpopulations at total mobility rate *δ*. Along a link, an individual in a subpopulation whose degree is *k* travels to another subpopulation of degree $k'$ at rate $d_{kk'}$, and $\delta=k\sum_{k'} P(k'|k)d_{kk'}$. When the mobility rate depends on traffic (passengers along the link),

1.2 $$ d_{kk'}=\delta\frac{w_{kk'}}{T_{k}}, $$

where $w_{kk'}$ represents the average traffic (passengers along the link node with degree *k* and node with degree $k'$ per day) on the link between two subpopulations with degrees *k* and $k'$, and behaves as

1.3 $$ w_{kk'}=w_{0}\bigl(kk' \bigr)^{\theta}, $$

here $w_{0}$ and *θ* are positive constants, $T_{k}$ is the total average traffic (passengers move out of node) of subpopulations with degree *k* (per day), and

1.4 $$ T_{k}=k\sum_{k'} P \bigl(k'|k\bigr)w_{kk'}. $$

Then Colizza and Vespignani established a model as follows:

1.5 $$ \dot{I_{k}}=-\delta I_{k}+(1-\delta)\biggl[-\mu I_{k}+\beta \frac{S_{k}I_{k}}{N_{k}}\biggr]+k\sum _{k'} P\bigl(k'|k\bigr)d_{k'k}\biggl[(1- \mu)I_{k'}+\beta \frac{S_{k'}I_{k'}}{N_{k'}}\biggr]. $$

As is known in the case of complex networks, the notation $P(k'|k)$ represents the conditional probability that any given edge departing from a node of degree *k* is pointing to a node of degree $k'$. Moreover, this work provided a good tool to calculate a global invasion threshold.

Applications of HMF theory have launched a variety of researches: factors including network structures [12–15], human mobility patterns [11, 16–18], human behaviors [19–22], human contact patterns [14, 23], travel restrictions [24], heterogeneous dwelling time in subpopulations [18], pathogen competition [25], and so forth, have been investigated. It has been shown that substrate network structures play an essential role in the spatial spread of infectious diseases [12, 13, 15]. In real-world networks, human mobility patterns vary in a very complicated way, e.g., large-scale air-travel (typical case [11]), small-scale recurrent visits of subpopulations (or commuting flows) [16–18, 26, 27], etc. Safety-driven people’s behavioral responses to the infectious diseases have also been found to be able to accelerate the infectious disease spread [19–22], contrary to willingness. With regard to human contact patterns, heterogeneous mixing [23] and subpopulations with network topology have been investigated.

For the results reported so far, the vast majority of researches are based on HMF theory [11]. According to (1.1), under the assumption that size of population in nodes with the same degree *k* stays the same, the equations

1.6 $$ \sum_{j\in A} S_{j}^{k}I_{j}^{k}=V_{k}^{*}S_{k}I_{k} $$

are equivalent to the case where the number of susceptible and infectious individuals in nodes whose degree are *k* are the same with each other, respectively. This conclusion holds when $V_{k}^{*}=1$. For $V_{k}^{*}=2$, with loss of generality, these two nodes get label 1 and 2, respectively. The number of susceptible and infectious individuals in node label 1 are $S_{1}^{k}$ and $I_{1}^{k}$. And there are $S_{2}^{k}$ susceptible individuals and $I_{2}^{k}$ infectious individuals in a node whose label is 2. From (1.6), we have

1.7 $$ S_{1}^{k}I_{1}^{k}+S_{2}^{k}I_{2}^{k}=2S_{k}I_{k}. $$

Substituting (1.1) into (1.7), and multiplying by 2 on the two sides of the above equations lead to

$$2\bigl(S_{1}^{k}I_{1}^{k}+S_{2}^{k}I_{2}^{k} \bigr)=\bigl(S_{1}^{k}+S_{2}^{k}\bigr) \bigl(I_{1}^{k}+I_{2}^{k}\bigr), $$

yielding

$$\bigl(S_{1}^{k}-S_{2}^{k}\bigr) \bigl(I_{1}^{k}-I_{2}^{k}\bigr)=0. $$

To make the equations hold, either $S_{1}^{k}=S_{2}^{k}$ or $I_{1}^{k}=I_{2}^{k}$, according to the assumption $S_{1}^{k}+I_{1}^{k}=N_{1}^{k}=N_{2}^{k}=S_{2}^{k}+I_{2}^{k}$, so both $S_{1}^{k}=S_{2}^{k}$ and $I_{1}^{k}=I_{2}^{k}$ are satisfied. For the case of $V_{k}^{*}>2$, for simplicity, label these $V_{k}^{*}$ nodes with integers $1,\ldots,V_{k}^{*}$, respectively, according to Eq. (1.6) and Eq. (1.1), we have

$$V_{k}^{*}\sum_{j=1}^{V_{k}^{*}} S_{j}^{k}I_{j}^{k}=\sum _{j=1}^{V_{k}^{*}} S_{j}^{k} \sum _{j=1}^{V_{k}^{*}} I_{j}^{k}. $$

We use the assumption that all nodes with the same degree *k* have the same number of individuals, i.e., $N_{i}^{k}=N_{j}^{k}, i,j=1,\ldots,V_{k}^{*}$. Replacing $S_{j}^{k}$ by $N_{j}^{k}-I_{j}^{k}$, and after some algebra, we have

$$\sum_{i=1}^{V_{k}^{*}} \bigl(V_{k}^{*}-1 \bigr) \bigl(I_{i}^{k}\bigr)^{2}-\sum _{i=1}^{V_{k}^{*}} I_{i}^{k}\sum _{j=1,j\neq i}^{V_{k}^{*}} I_{j}^{k}=0, $$

that is,

$$\sum_{i=1}^{V_{k}^{*}}\sum _{j=i+1}^{V_{k}^{*}} \bigl(I_{i}^{k}-I_{j}^{k} \bigr)^{2}=0. $$

Hence $I_{i}^{k}=I_{j}^{k}$, accordingly $S_{i}^{k}=S_{j}^{k}, i,j=1,\ldots,V_{k}^{*}$. From what has been discussed above, under the assumption that the number of individuals of all nodes with degree *k* keeps consistent, the number of susceptible individuals and infectious individuals of all nodes with degree *k*, respectively, being the same is a necessary condition for Eq. (1.6). Once $I_{i}^{k}\neq I_{j}^{k}$ for some *i* and *j*, Eq. (1.6) does not hold.

It is obvious that the HMF assumption in metapopulation network ignores heterogeneities in subpopulations with the same degree. Under this assumption, the total individuals, the numbers of susceptible individuals and infectious individuals in subpopulations with the same degree are always the same at any time, respectively, which is hard to achieve. Even though the size of a population in nodes with the same degree is identical, there may be a great difference for individuals in different states among nodes in the course of infectious disease spreading, resulting in error for forecasting infectious disease spreading. Motivated by reducing even eliminating this error, we take heterogeneities in subpopulations with the same degree into account, define the number of susceptible and infectious individuals in subpopulations with the same degree as a two-dimensional random variable and we propose a deterministic SIS model according to a continuous-time Markov chain (CTMC) on heterogeneous metapopulation networks to curve the global transmission of infections more precisely. The results show that our model extends and optimizes the HMF model in [11].

The paper is organized as follows. In Sect. 2, we firstly present necessary assumptions and propose a deterministic general model based on CTMC. Next, two moment closure models are given based on a two-dimensional constant distribution and a two-dimensional log-normal distribution in Sects. 3 and 4, respectively. Furthermore, in Sect. 5 we conduct numerical simulations on models and stochastic realizations. Conclusions and a discussion are given in Sect. 6. For simplicity, the definitions of abbreviations presented in the article are given in Table 1.

**Table 1:** Abbreviations in the article

Abbreviations	Full names
SARS	Severe Acute Respiratory Syndromes
MERS-CoV	Middle East respiratory syndrome coronavirus
SIS	susceptible-infectious-susceptible
HMF	heterogeneous mean-field
CTMC	continuous-time Markov chain
DFE	disease-free equilibrium

## The SIS infectious disease model based on CTMC

In this section, following the idea of stochastic process, we derive a deterministic SIS model on metapopulation network. The following assumptions will be used throughout the paper.

(H1): Global spread of infectious diseases is relatively fast timescales such that changes in demographics (e.g., births, aging, deaths) is negligible.
(H2): Network is connected and the minimal and maximal degrees are $k_{\mathrm{min}}$ and $k_{\mathrm{max}}$, respectively.
(H3): The memory of individuals for the origin is not taken into account.
(H4): For the Markov process, at each time step Δ*t*, the following four events occur simultaneously.
  (i) Disease transmission process: each susceptible individual is infected at transmission rate *β* by infectious individuals.
  (ii) Recovery process: every infectious individual recovers at rate *μ*.
  (iii) Mobility process consists of two processes: emigration and immigration. For a subpopulation with degree *k*, in emigration process, each individual in a subpopulation leaves this subpopulation at rate *δ* to its neighbor subpopulations; while in an immigration process, each individual in neighbor subpopulation with degree $k'$ travels to it at rate $d_{k'k}$.

### Model derivation

Before using CTMC to derive the deterministic model, in Table 2 we present the necessary notations for model parameters and model formation. To clarify the derivation of our models, the probability function is elucidated in what follows.

**Table 2:** Parameters description

Major parameters	Description
*M*	The maximum number of individuals among all subpopulations.
$(S^{k},I^{k})$	Two-dimensional random variable with the numbers of susceptible and infectious individuals in a subpopulation with degree *k*.
$V_{k}(s,i)(t)$	The number of nodes (subpopulations) with degree *k*, in which susceptible and infectious individuals are *s* and *i* at time *t*, respectively, *s*,*i*∈0,1,…,*M*.
$p_{s,i}^{k}(t)$	Probability that the numbers of susceptible and infectious individuals in a subpopulation with degree *k* are *s* and *i* at time *t*, respectively, that is, $p_{s,i}^{k}(t)=P\{(S^{k},I^{k})(t)=(s,i),S^{k},I^{k}\in{0,1,\ldots,M}\}$.
$p^{k}_{s+\Delta s,i+\Delta i}(\Delta t)$	The transition probability from state (*s*,*i*) to state (*s* + Δ*s*,*i* + Δ*i*) in the interval Δ*t* in a homogeneous Markov process.
$\langle f(s,i)\rangle_{k}$	The expectation of the function *f*(*s*,*i*), $\langle f(s,i)\rangle_{k}=\sum_{s,i} f(s,i)p_{s,i}^{k}$. Specially, $\langle s\rangle_{k}$ and $\langle i\rangle_{k}$ are expectations of susceptible individuals and infectious individuals, respectively.

The probability function with respect to susceptible and infectious individuals in a subpopulation with degree *k* is

$$ p_{s,i}^{k}(t)=P\bigl\{ \bigl(S^{k},I^{k} \bigr) (t)=(s,i)\bigr\} =\frac{V_{k}(s,i)(t)}{V_{k}^{*}}, $$

where $s=0,1,\ldots,M$, $i=0,1,\ldots,M-s$, and $\sum_{s,i} p_{s,i}^{k}(t)=1$. If $(s,i)$ lies outside of this range, the probability is assumed to be zero.

#### The stochastic process of two-dimensional random variable $(S^{k},I^{k})$

For this stochastic process, firstly, we derive the forward Kolmogorov differential equation about two-dimensional random variable $(S^{k},I^{k})$.

##### The forward Kolmogorov differential equation about $(S^{k},I^{k})$

Define the transition probability about $(S^{k},I^{k})$ from state $(S^{k},I^{k})(t)=(s,i)$ to state $(S^{k},I^{k})(t+\Delta t)=(s+\Delta s,i+\Delta i)$ in the time interval Δ*t* as

$$p^{k}_{s+\Delta s,i+\Delta i}(t+\Delta t,t)=P\bigl\{ \bigl(S^{k},I^{k} \bigr) (t+\Delta t)=(s+\Delta s,i+\Delta i)|\bigl(S^{k},I^{k} \bigr) (t)=(s,i))\bigr\} . $$

Note that, for a homogeneous Markov process, the transition probability does not depend on the starting time *t*, thus it is expressed as $p^{k}_{s+\Delta s,i+\Delta i}(\Delta t)$. In the paper, we assume the Markov process is homogeneous. Therefore, the transition probabilities about $(S^{k},I^{k})$ satisfy

2.1 $$ p^{k}_{s+\Delta s,i+\Delta i}(\Delta t)= \textstyle\begin{cases} \beta si \Delta t +o(\Delta t), &\Delta s=-1,\Delta i=1; \\ \mu i \Delta t +o(\Delta t), &\Delta s=1,\Delta i=-1; \\ \delta s \Delta t +o(\Delta t), &\Delta s=-1,\Delta i=0; \\ \delta i \Delta t +o(\Delta t), &\Delta s=0,\Delta i=-1; \\ k\sum_{k'}{\sum_{s',i'} P(k'|k)d_{k'k}s'\Delta t} +o(\Delta t),&\Delta s=1,\Delta i=0; \\ k\sum_{k'}{\sum_{s',i'} P(k'|k)d_{k'k}i'\Delta t} +o(\Delta t), &\Delta s=0,\Delta i=1; \\ 1-(\beta si +\mu i +\delta s +\delta i \\ \quad {}+k\sum_{k'}{\sum_{s',i'} P(k'|k)d_{k'k}s'}\\ \quad {}+k\sum_{k'}{\sum_{s',i'} P(k'|k)d_{k'k}i'})\Delta t +o(\Delta t), &\Delta s=0,\Delta i=0; \\ o(\Delta t),& \mbox{otherwise}, \end{cases} $$

in which the time step Δ*t* must be chosen sufficiently small

$$ \begin{aligned} &\max_{k\in \{k_{\mathrm{min}},\ldots,k_{\mathrm{max}}\}} \biggl\{ \biggl(\beta si +\mu i +\delta s +\delta i +k\sum_{k'}{\sum _{s',i'} P\bigl(k'|k\bigr)d_{k'k}s'} \\ &\quad {}+k\sum_{k'}{\sum_{s',i'} P\bigl(k'|k\bigr)d_{k'k}i'}\biggr)\Delta t \biggr\} \leq 1. \end{aligned} $$

Hence, we obtain the forward Kolmogorov equation as follows:

$$\begin{aligned} p_{s,i}^{k}(t+\Delta t)={}&\beta (s+1) (i-1)p_{s+1,i-1}^{k}(t) \Delta t +\mu (i+1)p_{s-1,i+1}^{k}(t) \Delta t \\ &{}+\delta(s+1)p_{s+1,i}^{k}(t) \Delta t +\delta(i+1)p_{s,i+1}^{k}(t) \Delta t \\ &{}+kp_{s-1,i}^{k}(t)\sum_{k'}{\sum _{s',i'}P\bigl(k'|k\bigr)d_{k'k} \bigl(s'+1\bigr)p_{s'+1,i'}^{k}(t)\Delta t} \\ &{}+kp_{s,i-1}^{k}(t)\sum_{k'}{\sum _{s',i'} P\bigl(k'|k\bigr)d_{k'k} \bigl(i'+1\bigr)p_{s',i'+1}^{k}(t)\Delta t} \\ &{}+p_{s,i}^{k}(t) \biggl\{ 1-\biggl(\beta si +\mu i +\delta s + \delta i +k\sum_{k'}{\sum _{s',i'}P\bigl(k'|k\bigr)d_{k'k}s'p_{s',i'}^{k}(t)} \\ &{}+k\sum_{k'}{\sum_{s',i'} P\bigl(k'|k\bigr)d_{k'k}i'p_{s',i'}^{k}(t)} \biggr)\Delta t \biggr\} +o(\Delta t). \end{aligned}$$

Subtracting $p_{s,i}^{k}(t)$ on both sides, dividing by Δ*t* and letting $\Delta t\to 0$ give rise to the forward Kolmogorov differential equation,

2.2 $$\begin{aligned} \frac{dp_{s,i}^{k}(t)}{dt}={}&\beta (s+1) (i-1)p_{s+1,i-1}^{k}(t) +\mu (i+1)p_{s-1,i+1}^{k}(t) \ \\ &{}+\delta(s+1)p_{s+1,i}^{k}(t) +\delta(i+1)p_{s,i+1}^{k}(t) \ \\ &{}+kp_{s-1,i}^{k}(t)\sum_{k'}{\sum _{s',i'}P\bigl(k'|k\bigr)d_{k'k} \bigl(s'+1\bigr)p_{s'+1,i'}^{k}(t)} \ \\ &{}+kp_{s,i-1}^{k}(t)\sum_{k'}{\sum _{s',i'} P\bigl(k'|k\bigr)d_{k'k} \bigl(i'+1\bigr)p_{s',i'+1}^{k}(t)} \ \\ &{}-p_{s,i}^{k}(t) \biggl\{ \beta si +\mu i +\delta s +\delta i +k\sum_{k'}{\sum_{s',i'}P \bigl(k'|k\bigr)d_{k'k}s'p_{s',i'}^{k}(t)} \ \\ &{}+k\sum_{k'}{\sum_{s',i'} P\bigl(k'|k\bigr)d_{k'k}i'p_{s',i'}^{k}(t)} \biggr\} . \end{aligned}$$

Now we calculate the expectations for susceptible and infectious individuals: $\langle s\rangle_{k}$ and $\langle i\rangle_{k}$ in subpopulations with degree *k*.

##### The deterministic equations of expectations $\langle s\rangle_{k},\langle i\rangle_{k}$

Further, we apply the obtained forward Kolmogorov differential equation (2.2) to derive the deterministic equations of $\langle s\rangle_{k},\langle i\rangle_{k}$, i.e., the rates of changes of the expectations $\langle s\rangle_{k},\langle i\rangle_{k}$ of $S^{k},I^{k}$, respectively.

Now, we give the deterministic equation of $\langle s\rangle_{k}$. Multiplying Eq. (2.2) by *s* and summing over *s* and *i*, yield

2.3 $$\begin{aligned} \frac{d\langle s\rangle_{k}(t)}{dt}={}&\beta \sum_{s,i}s(s+1) (i-1)p_{s+1,i-1}^{k}(t) +\mu \sum_{s,i}s(i+1)p_{s-1,i+1}^{k}(t) \\ &{}+\delta\sum_{s,i}s(s+1)p_{s+1,i}^{k}(t) +\delta\sum_{s,i}s(i+1)p_{s,i+1}^{k}(t) \\ &{}+k\sum_{s,i}sp_{s-1,i}^{k}(t)\sum _{k'}{\sum_{s',i'}P \bigl(k'|k\bigr)d_{k'k}\bigl(s'+1 \bigr)p_{s'+1,i'}^{k}(t)} \\ &{}+k\sum_{s,i}sp_{s,i-1}^{k}(t)\sum _{k'}{\sum_{s',i'} P \bigl(k'|k\bigr)d_{k'k}\bigl(i'+1 \bigr)p_{s',i'+1}^{k}(t)} \\ &{}-\sum_{s,i}sp_{s,i}^{k}(t) \biggl\{ \beta si +\mu i +\delta s +\delta i +k\sum_{k'}{ \sum_{s',i'}P\bigl(k'|k \bigr)d_{k'k}s'p_{s',i'}^{k}(t)} \\ &{}+k\sum_{k'}{\sum_{s',i'} P\bigl(k'|k\bigr)d_{k'k}i'p_{s',i'}^{k}(t)} \biggr\} . \end{aligned}$$

Simplifying Eq. (2.3), we obtain

2.4 $$\begin{aligned} \frac{d\langle s\rangle_{k}(t)}{dt}={}&{-}\beta \sum_{s,i}(s+1) (i-1)p_{s+1,i-1}^{k}(t) +\mu \sum_{s,i}(i+1)p_{s-1,i+1}^{k}(t) \\ &{}-\delta\sum_{s,i}(s+1)p_{s+1,i}^{k}(t) +k\sum_{k'}{\sum_{s',i'}P \bigl(k'|k\bigr)d_{k'k}\bigl(s'+1 \bigr)p_{s'+1,i'}^{k}(t)}. \end{aligned}$$

Substituting $\langle s\rangle_{k}=\sum_{s,i}sp_{s,i}^{k}$, $\langle i\rangle_{k}=\sum_{s,i}ip_{s,i}^{k}$ and $\langle si\rangle_{k}=\sum_{s,i}sip_{s,i}^{k}$ into Eq. (2.4), we get

2.5 $$ \frac{d\langle s\rangle_{k}}{dt}=-\beta \langle si\rangle_{k}+\mu \langle i\rangle_{k}-\delta\langle s\rangle_{k}+k\sum _{k'}{P\bigl(k'|k\bigr)d_{k'k}\langle s \rangle_{k'}}, $$

where $\langle si\rangle_{k}$ represents the average total number of all susceptible individuals contacting with infectious individuals in a subpopulation with degree *k*.

Applying the same approach, we give the deterministic equation about $\langle i\rangle_{k}$ as follows:

2.6 $$ \frac{d\langle i\rangle_{k}}{dt}=\beta \langle si\rangle_{k}-\mu \langle i\rangle_{k}-\delta\langle i\rangle_{k}+k\sum _{k'}{P\bigl(k'|k\bigr)d_{k'k}\langle i \rangle_{k'}}. $$

We therefore obtain the following deterministic model:

2.7a $$\begin{aligned} &\frac{d\langle s\rangle_{k}}{dt}=-\beta \langle si\rangle_{k}+\mu \langle i \rangle_{k}-\delta\langle s\rangle_{k}+k\sum _{k'}{P\bigl(k'|k\bigr)d_{k'k}\langle s \rangle_{k'}}, \end{aligned}$$

2.7b $$\begin{aligned} &\frac{d\langle i\rangle_{k}}{dt}=\beta \langle si\rangle_{k}-\mu \langle i \rangle_{k}-\delta\langle i\rangle_{k}+k\sum _{k'}{P\bigl(k'|k\bigr)d_{k'k}\langle i \rangle_{k'}}. \end{aligned}$$

For the sake of convenience, we call system (2.7a)–(2.7b) the general model. Obviously, the general model (2.7a)–(2.7b) is not closed. In order to make it closed, we need to derive the rate of changes of $\langle si\rangle_{k}$. Multiplying Eq. (2.2) by the product *si*, summing over *s* and *i* and simplifying yield

$$\begin{aligned} \frac{d\langle si\rangle_{k}}{dt}={}& \sum_{s,i}si\frac{dp_{s,i}^{k}}{dt}, \\ ={}&\beta \bigl\langle s^{2}i\bigr\rangle _{k}-\beta \bigl\langle si^{2}\bigr\rangle _{k}-(\beta+\gamma+2\delta) \langle si\rangle_{k}+\gamma \bigl\langle i^{2}\bigr\rangle _{k}-\gamma \langle i\rangle_{k} \\ &{}+k\langle i\rangle_{k} \sum_{k'} P \bigl(k'|k\bigr)d_{k'k}\langle s\rangle_{k'} +k \langle s\rangle_{k} \sum_{k'} P \bigl(k'|k\bigr)d_{k'k}\langle i\rangle_{k'}, \end{aligned}$$

here $\langle s^{2}i\rangle_{k}=\sum_{s,i}s^{2}i p_{s,i}^{k}$, and $\langle si^{2}\rangle_{k}=\sum_{s,i}si^{2} p_{s,i}^{k}$. The derivation above brings new parameters into play, $\langle i^{2}\rangle_{k}$, the average number of all infectious individuals contacting with infectious individuals in a subpopulation with degree *k*. In the same way, the rate of change of $\langle i^{2}\rangle_{k}$ reads

$$\begin{aligned} \frac{d\langle i^{2}\rangle_{k}}{dt}={}& \sum_{s,i}i^{2} \frac{dp_{s,i}^{k}}{dt}, \\ ={}&2\beta \bigl\langle si^{2}\bigr\rangle _{k}+\beta \langle si\rangle_{k}-2(\gamma+\delta) \bigl\langle i^{2}\bigr\rangle _{k} +(\gamma+\delta) \langle i\rangle_{k} \\ &{}+2k\langle i\rangle_{k} \sum_{k'} P \bigl(k'|k\bigr)d_{k'k}\langle i\rangle_{k'} +k \langle s\rangle_{k} \sum_{k'} P \bigl(k'|k\bigr)d_{k'k}\langle i\rangle_{k'}. \end{aligned}$$

So far, the model is still not closed because of the appearance of the third-order cumulants: $\langle s^{2}i\rangle_{k}$ and $\langle si^{2}\rangle_{k}$. Notice that calculation of third-order cumulants may bring new variables into play: fourth-order cumulants, and so on. Approaching the third-order cumulants by first- and second-order cumulants becomes necessary. It is clear that with different two-dimensional quasi-stationary distributions of $p_{s,i}^{k}$, the closed models vary. We will present two main moment closure equations in Sects. 3 and 4.

### Model analysis

Although the general model (2.7a)–(2.7b) is not closed, there exists a common property. Notice that the average total number of individuals in a subpopulation with degree *k* satisfies

$$N_{k}=\langle s\rangle_{k}+\langle i\rangle_{k}. $$

Summing (2.7a) and (2.7b), we have

2.8 $$ \frac{dN_{k}}{dt}=-\delta N_{k}+k\sum _{k'} P\bigl(k'|k\bigr)d_{k'k}N_{k'}. $$

In the paper, we overlook the degree correlations, that is to say, $P(k'|k)=k'P(k')/\langle k\rangle$. Let

$$\bar{N}=\sum_{k'} P\bigl(k' \bigr)N_{k'}. $$

Equation (2.8) becomes

2.9 $$ \frac{dN_{k}}{dt}=-\delta N_{k}+\delta \frac{k^{1+\theta}}{\langle k^{1+\theta}\rangle}\bar{N}, $$

where *N̄*, which is a positive constant, represents the average number of individuals in a subpopulation. It is obvious that Eqs. (2.9) have a unique globally asymptotically stable equilibrium $N_{k}^{*}=\frac{k^{1+\theta}}{\langle k^{1+\theta}\rangle}\bar{N}, k=k_{\mathrm{min}},\ldots,k_{\mathrm{max}}$.

## Moment closure based on two-dimensional constant distribution

We first present a closed model based on a two-dimensional constant distribution.

### Derivation of moment closure model based on two-dimensional constant distribution

For a fixed degree *k*, when the quasi-stationary of $p_{s,i}^{k}$ approaches a two-dimensional constant distribution, we have $\langle si\rangle_{k}=\langle s\rangle_{k}\langle i\rangle_{k}$, so the closed model is

3.1a $$\begin{aligned} &\frac{d\langle s\rangle_{k}}{dt}=-\beta \langle s\rangle_{k}\langle i \rangle_{k}+\mu \langle i\rangle_{k}-\delta \langle s \rangle_{k}+k\sum_{k'}{P \bigl(k'|k\bigr)d_{k'k}\langle s\rangle_{k'}}, \end{aligned}$$

3.1b $$\begin{aligned} &\frac{d\langle i\rangle_{k}}{dt}=\beta \langle s\rangle_{k}\langle i \rangle_{k}-\mu \langle i\rangle_{k}-\delta \langle i \rangle_{k}+k\sum_{k'}{P \bigl(k'|k\bigr)d_{k'k}\langle i\rangle_{k'}}. \end{aligned}$$

#### Remark 3.1

The assumption of two-dimensional constant distribution is equivalent to HMF assumption. Under the supposition that mobility process and disease transmission process occur simultaneously, our model (3.1a)–(3.1b) and the models in [11] are equivalent.

In order to study the asymptotic behavior of model, we consider the limiting system of (3.1a)–(3.1b),

3.2 $$ \frac{d\langle i\rangle_{k}}{dt}=\beta \bigl(N_{k}^{*}-\langle i \rangle_{k}\bigr)\langle i\rangle_{k}-\mu \langle i \rangle_{k}-\delta \langle i\rangle_{k}+k\sum _{k'}{P\bigl(k'|k\bigr)d_{k'k}\langle i \rangle_{k'}}. $$

For simplicity, we call (3.2) the moment closure model I.

### Model analysis

#### Global basic reproduction number

Firstly, we deduce global basic reproduction number by the approach in van den Driessche and Watmough [28]. Note that model (3.2) admits a unique disease-free equilibrium (DFE) $E^{0}=(\overbrace{0,\ldots,0}^{n}),n=k_{\mathrm{max}}-k_{\mathrm{min}}+1$. In the DFE $E^{0}$, the rate of appearance of new infections *F* and the rate of transfer of individuals out of the compartments *V* are given by

$$ F= \begin{pmatrix} f_{11} & f_{12} & \cdots & f_{1n}\\ f_{21} & f_{22} & \cdots & f_{2n}\\ \vdots & \vdots & \ddots & \vdots \\ f_{n1} & f_{n2} & \cdots & f_{nn} \end{pmatrix} $$

and

$$ V=(\mu+\delta)E_{n}, $$

where

$$ f_{ij}= \textstyle\begin{cases} \beta N_{(i+k_{\mathrm{min}}-1)}^{*}+\delta\frac{(i+k_{\mathrm{min}}-1)^{(1+\theta)}}{\langle k^{(1+\theta)}\rangle} P(i+k_{\mathrm{min}}-1), &i=j, \\ \delta\frac{(i+k_{\mathrm{min}}-1)^{(1+\theta)}}{\langle k^{(1+\theta)}\rangle} P(j+k_{\mathrm{min}}-1),&i\neq j, \end{cases} $$

here $i,j\in{1,\ldots,n}$.

Thus

$$FV^{-1}=\frac{1}{(\mu+\delta)}F. $$

Using the next-generation matrix [28], the global basic reproduction number is $R_{0}=\rho(FV^{-1})$, the spectral radius of the matrix $FV^{-1}$.

#### Global stability of disease-free equilibrium

Since $\langle i\rangle_{k}\in[0,N_{k}^{*}]$ for $k=k_{\mathrm{min}},\ldots,k_{\mathrm{max}}$, we study system (3.2) in $\Omega_{n}=\prod_{k=k_{\mathrm{min}}}^{k_{\mathrm{max}}} [0,N_{k}^{*}]$.

##### Lemma 3.1

*The set* $\Omega_{n}$ *is positively invariant for system* (3.2).

##### Theorem 3.1

*For system* (3.2), *if* $R_{0}<1$, *DFE* $E^{0}$ *is globally attractive in* $\Omega_{n}$.

##### Proof

To complete the proof, it is sufficient to show that

$$\lim_{t \to +\infty} \langle i\rangle_{k}=0,\quad k=k_{\mathrm{min}},\ldots,k_{\mathrm{max}}. $$

For system (3.2) with $\langle i\rangle_{k}\leq N_{k}^{*}$, we can obtain the inequality group

3.3 $$ \frac{d\langle i\rangle_{k}}{dt} \leq \beta N_{k}^{*}\langle i \rangle_{k}-\mu \langle i\rangle_{k}-\delta \langle i \rangle_{k}+k\sum_{k'}{P \bigl(k'|k\bigr)d_{k'k}\langle i\rangle_{k'}}. $$

Define an auxiliary linear system

3.4 $$ \frac{d\langle i\rangle_{k}}{dt} =\beta N_{k}^{*}\langle i \rangle_{k}-\mu \langle i\rangle_{k}-\delta \langle i \rangle_{k}+k\sum_{k'}{P \bigl(k'|k\bigr)d_{k'k}\langle i\rangle_{k'}}. $$

The coefficient matrix of the above system is $F-V$. When $R_{0}=\rho(FV^{-1})<1$, all eigenvalues of $F-V$ lie in the left half plane. Thus each non-negative solution of (3.3) satisfies

$$\lim_{t \to +\infty} \langle i\rangle_{k}=0,\quad k=k_{\mathrm{min}},\ldots,k_{\mathrm{max}}. $$

This implies that the zero solution of (3.4) is globally asymptotically stable. By comparison, each non-negative solution of (3.2) satisfies

$$ \lim_{t \to +\infty} \langle i\rangle_{k}=0, \quad k=k_{\mathrm{min}},\ldots,k_{\mathrm{max}}. $$

Accordingly, DFE $E^{0}$ of system (3.2) is globally attractive. □

#### Global stability of endemic equilibrium

Next, we analyze the existence and global stability of endemic equilibrium.

##### Theorem 3.2

*If* $R_{0}>1$, *system* (3.2) *admits a unique endemic equilibrium* $E^{*}=(\langle i\rangle_{k_{\mathrm{min}}}^{*},\ldots, \langle i\rangle_{k_{\mathrm{max}}}^{*})$ *which is globally asymptotically stable with respect to any initial value* $y(0)\in \Omega_{n}-\{0\}$:=$\Omega_{n}^{+}$.

##### Proof

We will use the theory of cooperate system in Corollary 3.2 in [29] to prove the existence and global stability of endemic equilibrium.

In fact, let $f:\Omega_{n}^{+} \rightarrow \Omega_{n}$ be defined by the right-hand side of (3.2), $f=(f_{k_{\mathrm{min}}},\ldots,f_{k_{\mathrm{max}}})$. Clearly *f* is continuously differentiable, $f(0)=0$, $f_{i}(y) \geq 0$ for all *y* $(=(\langle i\rangle_{k_{\mathrm{min}}},\ldots,\langle i\rangle_{k_{\mathrm{max}}})) \in \Omega_{n}^{+}$ with $y_{i}=0$ and $\partial f_{i} / \partial y_{j} \geq 0$, $i \neq j$ for $y\in \Omega_{n}^{+}$. So *f* is cooperative. Clearly $Dy=(\partial f_{i} / \partial y_{j})_{k_{\mathrm{min}}\leq i,j \leq k_{\mathrm{max}}}$ is irreducible for every $y\in \Omega_{n}^{+}$.

Note that, for $\forall \alpha \in (0,1)$ and $y_{i}>0$,

$$\begin{aligned} f_{i}(\alpha y)&=\alpha \biggl\{ \beta \bigl(N_{k}^{*}-\alpha \langle i\rangle_{k}\bigr)\langle i\rangle_{k}- \mu \langle i \rangle_{k}-\delta \langle i\rangle_{k}+k\sum _{k'}{P\bigl(k'|k\bigr)d_{k'k}\langle i \rangle_{k'}}\biggr\} \\ &\geq \alpha \biggl\{ \beta \bigl(N_{k}^{*}-\langle i \rangle_{k}\bigr)\langle i\rangle_{k}-\mu \langle i \rangle_{k}-\delta \langle i\rangle_{k}+k\sum _{k'}{P\bigl(k'|k\bigr)d_{k'k}\langle i \rangle_{k'}}\biggr\} \\ &=\alpha f_{i}(y). \end{aligned}$$

Thus *f* is strong sublinear on $\Omega_{n}^{+}$. By Lemma 2 and Corollary 3.2 in [29], we conclude that system (3.2) admits a unique endemic equilibrium $E^{*}=(\langle i\rangle_{k_{\mathrm{min}}}^{*},\ldots,\langle i\rangle_{k_{\mathrm{max}}}^{*})$ which is globally asymptotically stable. □

## Moment closure based on two-dimensional log-normal distribution

Following Keeling [30] we assume that the quasi-stationary distribution is approximately two-dimensional log-normal. The calculation of all cumulants of orders greater than first is performed by considering all possible pairwise combinations of the elements and multiplying by the appropriate moment. In this case,

4.1 $$ { } \langle si\rangle_{k}=\langle s\rangle_{k} \langle i\rangle_{k}\xi_{k}=\sum_{s,i} si p_{s,i}^{k}, $$

where $\xi_{k}$ is the multiplicative covariance between infectious and susceptible individuals within a subpopulation with degree *k*. Taking derivatives by *t* at both ends of the second equality at the same time, transposition, substitution and simplification, leads to

$$\begin{aligned} \langle s\rangle_{k}\langle i\rangle_{k}\frac{d\xi_{k}}{dt} ={}& \sum_{s,i}si\frac{dp_{s,i}^{k}}{dt}-\langle s \rangle_{k}\xi_{k}\frac{d\langle i\rangle_{k}}{dt}-\langle i \rangle_{k}\xi_{k}\frac{d\langle s\rangle_{k}}{dt} \\ ={}&\beta \langle s\rangle_{k}^{2}\langle i \rangle_{k}\xi_{k}^{2}(\hat{V_{\langle s\rangle_{k}}}+1) - \beta \langle s\rangle_{k}\langle i\rangle_{k}^{2} \xi_{k}^{2}(\hat{V_{\langle i\rangle_{k}}}+1) \\ &{}-(\beta+2\mu+2\delta) \langle s\rangle_{k}\langle i \rangle_{k}\xi_{k}-\mu \langle i\rangle_{k} \\ &{}+\bigl(\mu \hat{V_{\langle i\rangle_{k}}}+\mu \langle s\rangle_{k}+\delta \xi_{k}\bigr)\langle i\rangle_{k}^{2}+\delta\langle s\rangle_{k}^{2}\xi_{k} \\ &{}+k\bigl(\langle i\rangle_{k}-\langle s\rangle_{k} \xi_{k}\bigr) \sum_{k'} P \bigl(k'|k\bigr)d_{k'k}\langle s\rangle_{k'} \\ &{}+k\bigl(\langle s\rangle_{k}-\langle i\rangle_{k} \xi_{k}\bigr) \sum_{k'} P \bigl(k'|k\bigr)d_{k'k}\langle i\rangle_{k'}. \end{aligned}$$

This brings in two other parameters, $\hat{V_{\langle s\rangle_{k}}}$ and $\hat{V_{\langle i\rangle_{k}}}$, the variance in susceptibles and infectious within a subpopulation with degree *k*. In a similar way, we obtain

$$\begin{aligned} \langle s\rangle_{k}^{2}\frac{d\hat{V_{\langle s\rangle_{k}}}}{dt} ={}& \sum _{s,i}s^{2}\frac{dp_{s,i}^{k}}{dt}-2\langle s \rangle_{k}\hat{V_{\langle s\rangle_{k}}}\frac{d\langle s\rangle_{k}}{dt} \\ ={}&{-}2\beta \langle s\rangle_{k}^{2}\langle i \rangle_{k}\hat{V_{\langle s\rangle_{k}}}\xi_{k}(\xi_{k}-1) +\beta \langle s\rangle_{k}\langle i\rangle_{k} \xi_{k} +\mu \langle i\rangle_{k}+\delta\langle s \rangle_{k} \\ &{}+2\mu \langle s\rangle_{k}\langle i\rangle_{k}( \xi_{k}-\hat{V_{\langle s\rangle_{k}}}) +2k\langle s\rangle_{k}(1- \hat{V_{\langle s\rangle_{k}}}) \sum_{k'} P \bigl(k'|k\bigr)d_{k'k}\langle s\rangle_{k'} \\ &{}+k\sum_{k'} P\bigl(k'|k \bigr)d_{k'k}\langle s\rangle_{k'} \end{aligned}$$

and

$$\begin{aligned} \langle i\rangle_{k}^{2}\frac{d\hat{V_{\langle i\rangle_{k}}}}{dt} ={}& \sum _{s,i}i^{2}\frac{dp_{s,i}^{k}}{dt}-2\langle i \rangle_{k}\hat{V_{\langle i\rangle_{k}}}\frac{d\langle i\rangle_{k}}{dt} \\ ={}&2\beta \langle s\rangle_{k}\langle i\rangle_{k}^{2} \hat{V_{\langle i\rangle_{k}}}\xi_{k}(\xi_{k}-1) +\beta \langle s \rangle_{k}\langle i\rangle_{k}\xi_{k} +(\mu+\delta) \langle i\rangle_{k} \\ &{}+2k\langle i\rangle_{k}(1-\hat{V_{\langle i\rangle_{k}}}) \sum _{k'} P\bigl(k'|k\bigr)d_{k'k}\langle i \rangle_{k'} +k\sum_{k'} P \bigl(k'|k\bigr)d_{k'k}\langle i\rangle_{k'}. \end{aligned}$$

From the above derivation, we obtain a moment closure model based on the log-normal distribution

4.2a $$\begin{aligned} &\frac{d\langle s\rangle_{k}}{dt}=-\beta \langle s\rangle_{k}\langle i \rangle_{k}\xi_{k}+\mu \langle i\rangle_{k}-\delta \langle s\rangle_{k}+k\sum_{k'}{P \bigl(k'|k\bigr)d_{k'k}\langle s\rangle_{k'}}, \end{aligned}$$

4.2b $$\begin{aligned} &\frac{d\langle i\rangle_{k}}{dt}=\beta \langle s\rangle_{k}\langle i \rangle_{k}\xi_{k}-\mu \langle i\rangle_{k}-\delta \langle i\rangle_{k}+k\sum_{k'}{P \bigl(k'|k\bigr)d_{k'k}\langle i\rangle_{k'}}, \end{aligned}$$

4.2c $$\begin{aligned} &\begin{aligned}[b] \langle s\rangle_{k}\langle i\rangle_{k}\frac{d\xi_{k}}{dt} ={}&\beta \langle s\rangle_{k}^{2}\langle i \rangle_{k}\xi_{k}^{2}(\hat{V_{\langle s\rangle_{k}}}+1) - \beta \langle s\rangle_{k}\langle i\rangle_{k}^{2} \xi_{k}^{2}(\hat{V_{\langle i\rangle_{k}}}+1) \\ &{}-(\beta+2\mu+2\delta) \langle s\rangle_{k}\langle i \rangle_{k}\xi_{k} +\bigl(\mu \hat{V_{\langle i\rangle_{k}}}+\mu \langle s\rangle_{k}+\delta\xi_{k}\bigr)\langle i \rangle_{k}^{2} \\ &{}+\delta\langle s\rangle_{k}^{2}\xi_{k}-\mu \langle i\rangle_{k} +k\bigl(\langle i\rangle_{k}-\langle s \rangle_{k}\xi_{k}\bigr) \sum_{k'} P\bigl(k'|k\bigr)d_{k'k}\langle s\rangle_{k'} \\ &{}+k\bigl(\langle s\rangle_{k}-\langle i\rangle_{k} \xi_{k}\bigr) \sum_{k'} P \bigl(k'|k\bigr)d_{k'k}\langle i\rangle_{k'}, \end{aligned} \end{aligned}$$

4.2d $$\begin{aligned} & \begin{aligned}[b] \langle s\rangle_{k}^{2}\frac{d\hat{V_{\langle s\rangle_{k}}}}{dt} ={}&{-}2\beta \langle s\rangle_{k}^{2}\langle i\rangle_{k} \hat{V_{\langle s\rangle_{k}}}\xi_{k}(\xi_{k}-1) +\beta \langle s \rangle_{k}\langle i\rangle_{k}\xi_{k} +\mu \langle i\rangle_{k}+\delta\langle s\rangle_{k} \\ &{}+2\mu \langle s\rangle_{k}\langle i\rangle_{k}( \xi_{k}-\hat{V_{\langle s\rangle_{k}}})+k\sum_{k'} P\bigl(k'|k\bigr)d_{k'k}\langle s\rangle_{k'} \\ &{}+2k\langle s\rangle_{k}(1-\hat{V_{\langle s\rangle_{k}}}) \sum _{k'} P\bigl(k'|k\bigr)d_{k'k}\langle s \rangle_{k'}, \end{aligned} \end{aligned}$$

4.2e $$\begin{aligned} &\begin{aligned}[b] \langle i\rangle_{k}^{2}\frac{d\hat{V_{\langle i\rangle_{k}}}}{dt} ={}&2\beta \langle s\rangle_{k}\langle i\rangle_{k}^{2} \hat{V_{\langle i\rangle_{k}}}\xi_{k}(\xi_{k}-1) +\beta \langle s \rangle_{k}\langle i\rangle_{k}\xi_{k} +(\mu+\delta) \langle i\rangle_{k} \\ &{}+2k\langle i\rangle_{k}(1-\hat{V_{\langle i\rangle_{k}}}) \sum _{k'} P\bigl(k'|k\bigr)d_{k'k}\langle i \rangle_{k'} \\ &{}+k\sum_{k'} P\bigl(k'|k \bigr)d_{k'k}\langle i\rangle_{k'}. \end{aligned} \end{aligned}$$

Accordingly, we have the following limiting system:

4.3a $$\begin{aligned} & \frac{d\langle i\rangle_{k}}{dt}= \beta \bigl(N_{k}^{*}-\langle i\rangle_{k} \bigr)\langle i\rangle_{k}\xi_{k}-\mu \langle i \rangle_{k}-\delta\langle i\rangle_{k} +k\sum_{k'}{P\bigl(k'|k \bigr)d_{k'k}\langle i\rangle_{k'}}, \end{aligned}$$

4.3b $$\begin{aligned} &\begin{aligned}[b] \bigl(N_{k}^{*}-\langle i\rangle_{k}\bigr)\langle i \rangle_{k}\frac{d\xi_{k}}{dt} ={}&\beta \bigl(N_{k}^{*}-\langle i \rangle_{k}\bigr)^{2}\langle i\rangle_{k} \xi_{k}^{2}(\hat{V_{\langle s\rangle_{k}}}+1)+\delta \bigl(N_{k}^{*}-\langle i\rangle_{k}\bigr)^{2} \xi_{k} \\ &{}-\beta \bigl(N_{k}^{*}-\langle i\rangle_{k}\bigr)\langle i \rangle_{k}^{2}\xi_{k}^{2}( \hat{V_{\langle i\rangle_{k}}}+1)-\mu \langle i\rangle_{k} \\ &{}-(\beta+2\mu+2\delta) \bigl(N_{k}^{*}-\langle i\rangle_{k} \bigr)\langle i\rangle_{k}\xi_{k} \\ &{}+\bigl[\mu \hat{V_{\langle i\rangle_{k}}}+\mu \bigl(N_{k}^{*}-\langle i \rangle_{k}\bigr) +\delta\xi_{k}\bigr]\langle i \rangle_{k}^{2} \\ &{}+k\bigl[\langle i\rangle_{k}-\bigl(N_{k}^{*}-\langle i \rangle_{k}\bigr)\xi_{k}\bigr]\sum _{k'} P\bigl(k'|k\bigr)d_{k'k} \bigl(N_{k'}^{*}-\langle i\rangle_{k'}\bigr) \\ &{}+k\bigl(N_{k}^{*}-\langle i\rangle_{k}-\langle i \rangle_{k}\xi_{k}\bigr) \sum_{k'} P\bigl(k'|k\bigr)d_{k'k}\langle i\rangle_{k'}, \end{aligned} \end{aligned}$$

4.3c $$\begin{aligned} & \begin{aligned}[b] \bigl(N_{k}^{*}-\langle i\rangle_{k}\bigr)^{2} \frac{d\hat{V_{\langle s\rangle_{k}}}}{dt} ={}&{-}2\beta \bigl(N_{k}^{*}-\langle i \rangle_{k}\bigr)^{2}\langle i\rangle_{k} \hat{V_{\langle s\rangle_{k}}}\xi_{k}(\xi_{k}-1) +\beta \bigl(N_{k}^{*}-\langle i\rangle_{k}\bigr)\langle i \rangle_{k}\xi_{k} \\ &{}+\mu \langle i\rangle_{k}+\delta\bigl(N_{k}^{*}-\langle i \rangle_{k}\bigr) +2\mu \bigl(N_{k}^{*}-\langle i \rangle_{k}\bigr)\langle i\rangle_{k}(\xi_{k}- \hat{V_{\langle s\rangle_{k}}}) \\ &{}+2k\bigl(N_{k}^{*}-\langle i\rangle_{k}\bigr) (1- \hat{V_{\langle s\rangle_{k}}}) \sum_{k'} P \bigl(k'|k\bigr)d_{k'k}\bigl(N_{k'}^{*}-\langle i \rangle_{k'}\bigr) \\ &{}+k\sum_{k'} P\bigl(k'|k \bigr)d_{k'k}\bigl(N_{k'}^{*}-\langle i\rangle_{k'} \bigr), \end{aligned} \end{aligned}$$

4.3d $$\begin{aligned} &\begin{aligned}[b] \langle i\rangle_{k}^{2}\frac{d\hat{V_{\langle i\rangle_{k}}}}{dt} ={}&2\beta \bigl(N_{k}^{*}-\langle i\rangle_{k}\bigr)\langle i \rangle_{k}^{2}\hat{V_{\langle i\rangle_{k}}}\xi_{k}( \xi_{k}-1) +\beta \bigl(N_{k}^{*}-\langle i\rangle_{k} \bigr)\langle i\rangle_{k}\xi_{k} \\ &{}+(\mu+\delta) \langle i\rangle_{k}+2k\langle i\rangle_{k}(1- \hat{V_{\langle i\rangle_{k}}}) \sum_{k'} P \bigl(k'|k\bigr)d_{k'k}\langle i\rangle_{k'} \\ &{}+k\sum_{k'} P\bigl(k'|k \bigr)d_{k'k}\langle i\rangle_{k'}. \end{aligned} \end{aligned}$$

For simplicity, we denote it as moment closure model II.

## Numerical results

In this section, we conduct extensive numerical simulations, stochastic simulations, and compare them. Numerical simulations of models are obtained by the fourth-order Runge–Kutta algorithm on MATLAB. We report stochastic simulation results from Monte Carlo simulations in a variety of different realizations on FORTRAN. Metapopulation networks are generated with the uncorrelated scale-free network model [31, 32] with *V* ranging from 100 to 1000 following the power-law degree distribution $P(k)\sim k^{-\gamma}$, $2<\gamma\leq3$ with minimum degree $k_{\mathrm{min}}=2$ and maximum degree $k_{\mathrm{max}}\leq V^{1/2}$. According to Ref. [33], $w_{0}=1$ and $\theta=0.5$. Simulation results are based on averaging over at least 50 realizations for initial conditions and network structures.

### Stability of closed models

First of all, focusing on stability of equilibria, we develop a series of numerical simulations about models (3.2) and (4.3a)–(4.3d) to discuss stability of systems.

#### Stability of equilibria of moment closure model I

**Figure 2:**
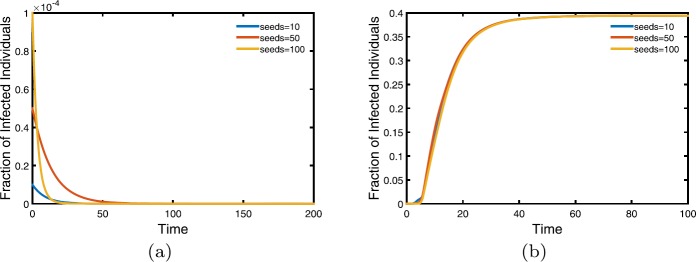
Evolution of the moment closure model I with the varying initial infectious individuals. Here we take $\mu=0.3$, $\gamma=2.1$, $\bar{N}=1000$, $V=1000$, $\delta=0.01$. Three lines represent different initial infectious seeds in a node chosen randomly. (**a**) $\beta=2e^{-5}$; (**b**) $\beta=2e^{-4}$

We present numerical integration of model (3.2) under two cases: $R_{0}<1$ and $R_{0}>1$ to investigate how the spread of infectious diseases depend on the threshold $R_{0}$ and whether the system stable or not. In Fig. 2, when $\beta=2e^{-5}$, $R_{0}$ is $0.9177<1$ (see Fig. 2(a)), while $R_{0}$ becomes $9.1729>1$ for $\beta=2e^{-4}$ (see Fig. 2(b)). We find that when $R_{0}<1$ the number of infectious individuals all approach zero under different initial conditions. Besides, the fraction of infectious individuals approaches 0.394 while $R_{0}>1$.

#### Stability of equilibrium of moment closure model II

**Figure 3:**
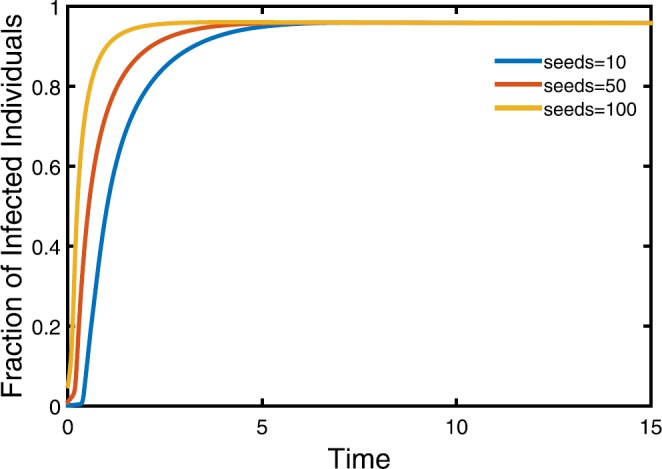
Evolution of the moment closure model II with the varying initial infectious individuals. Here we take $\beta=2e^{-4}$. Three lines represent different initial seeds every node. The other parameters are the same with Fig. 2

We further study system (4.3a)–(4.3d). As a system of weighted ordinary differential equations, it is unreasonable to perform $\langle i\rangle_{k}=0$. For simplicity, we assume that every node has infectious seeds at initial moments. In Fig. 3, the density of infectious individuals approaching a constant under different initial conditions, approximately 0.96, means that there exists an endemic equilibrium for system (4.3a)–(4.3d).

### Time courses of the density of infectious individuals

**Figure 4:**
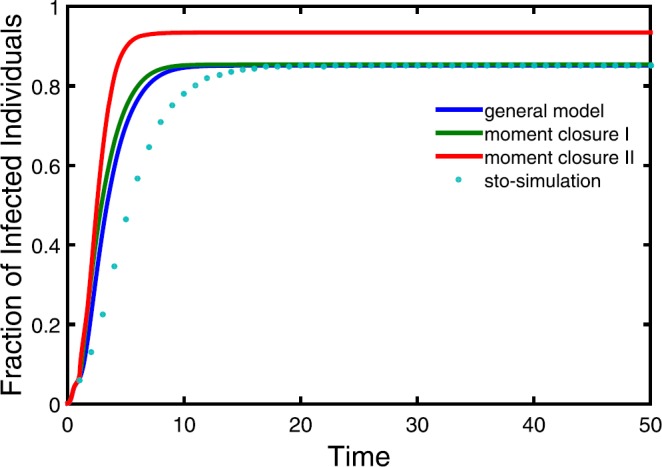
Comparison of models and stochastic simulations. In all cases, seed 10 infectious individuals in subpopulation with the maximum degree in the initial time. Here we take $\beta=2e^{-2}$, $\mu=0.3$, $\gamma=2.63$, $\delta=0.1$, $\bar{N}=100$, $V=100$

According to the forward Kolmogorov differential equation (2.2), we put forward model (2.6) to address the spread of infectious diseases on metapopulation networks. Further, to make the model (2.6) closed, we derive models (3.2) and (4.3a)–(4.3d). In Fig. 4, we compare these models with each other based on numerical simulations, and make a comparison with stochastic simulations at the same time. Red line, green line and blue line are calculated from the general model (2.6) coupling with (2.2), moment closure model I (3.2) and moment closure model II (4.3a)–(4.3d), respectively, while dots are averaging over 100 stochastic realizations. It is showed that the steady state of moment closure model II is higher than the others. Hence the assumption of a two-dimensional log-normal distribution is inappropriate for metapopulation networks. In the steady state, general model I fits perfectly with stochastic simulations, and moment closure model I takes the second place. For an early stage of infectious diseases, they overestimate the spreading of diseases on different levels. Overall, the general model extends and optimizes the HMF model.

### The effects of total mobility rate on disease transmission

**Figure 5:**
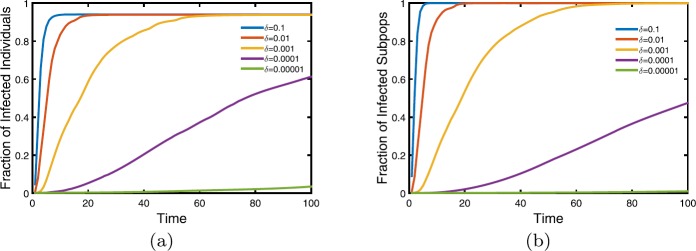
The impacts of total mobility rate on disease transmission. Five magnitudes of total mobility rate are performed. From bottom to top, increase one magnitude in turn. The left panel shows the fraction of individual infectious evolving over time, while the right panel is the proportion of infectious subpopulations. In detail, $\beta=5e^{-3}$ and other parameters are the same with Fig. 2. The values are obtained by averaging over 50 stochastic realizations

**Figure 6:**
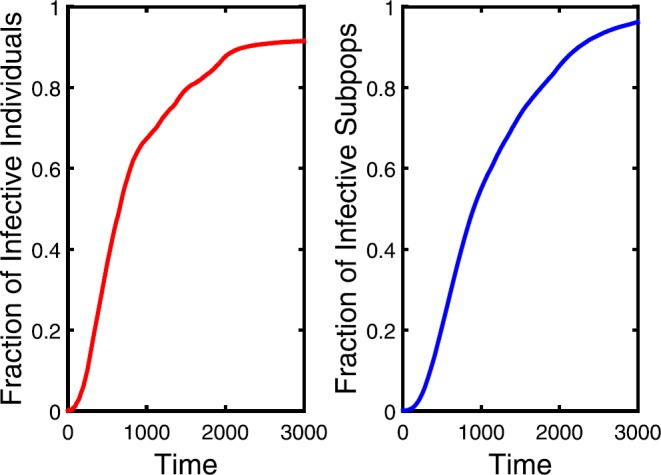
The density of infectious individuals for $\delta=1e^{-5}$. The other parameters are the same with Fig. 5

Finally, we discuss the impacts of total mobility rate on disease transmission and perform five magnitudes of total mobility rate. In Fig. 5, when $\delta=0.1$, diseases globally spread and rapidly achieve steady state. Then with the decrease of total mobility rate, the transmission slows down. When *δ* reduces to 0.00001, diseases hardly spread in a short time. Instead, in a long time, $t=3000$ or longer, infectious diseases will spread to the entire network as in Fig. 6. It is worth noticing that there is no impact of mobility rate on the steady of whole metapopulation network. So travel restriction in the early stage is an effective prevention and control of infectious diseases, and restriction must be timely and thoroughly, prohibiting travel over a period of time, for example.

## Conclusions and discussion

Following CTMC, we have derived a deterministic unclosed general model to portray disease transmission on metapopulation networks in which the heterogeneity of susceptible and infectious individuals in subpopulations with the same degree was considered. Then we closed the general model under the assumption of a two-dimensional constant distribution and two-dimensional log-normal distribution, respectively. And the existence and global stability of each of feasible equilibria of the system, which is based on two-dimensional constant distribution, have been proved mathematically and illustrated by numerical simulations. It has been shown by simulations that the general model we derived generalizes and optimizes the HMF model. In the study of the effects of total mobility rate on infection spread, we have seen that total mobility rate has a huge impact on disease transmission speed, but it has no effect on the steady state of infections. It is worth noting that even for a relatively small total mobility rate, diseases will spread globally as long as time is long enough. Therefore, timely and comprehensive travel restrictions are critical for disease control and prevention.

Although we have generalized and optimized the HMF model, there are still some problems in this paper to be further solved.

1. The deterministic general model extends and optimizes the HMF model in [11], however, numerical simulations did not fit perfectly the stochastic simulations before the transmission was up to steady. Hence, our model needs to be further improved.
2. We conducted numerical simulations for the moment closure II, and we found this system has an endemic equilibrium. It is interesting to verify the existence and global stability of the endemic equilibrium mathematically.
3. On the edge weights selection for metapopulation network, we supposed that weights were only dependent on the degrees of subpopulations. However, a real situation may be more complex, and weights may be the comprehensive outcome of populations, degrees and distances. For the total mobility rate, it is more appropriate to vary with the states of individuals.
